# Interethnic Workplace Conflict: Reciprocal Perception of Italian and Immigrant Blue-Collar Coworkers

**DOI:** 10.5964/ejop.2395

**Published:** 2022-05-31

**Authors:** Maria Grazia Monaci

**Affiliations:** 1 Department of Human and Social Sciences, University of Valle d’Aosta, Aosta, Italy; Open University, Milton Keynes, United Kingdom

**Keywords:** perceived conflict, interethnic contact, organizational identification, stereotype content

## Abstract

Past research has examined the beneficial effects of contact focusing mainly on the affective dimension of intergroup relationships. Limited research has examined cognitive dimensions, in particular considering at the same time minority and majority group perspectives. This study tested whether contact in a work context relates differentially to the perception of interethnic conflict in Italian (n = 67) and immigrant (n = 40, all male) blue-collar coworkers with the potential mediation of reciprocal stereotypical content (competence and warmth dimensions), interethnic attitudes, perceived discrimination, and whether organizational identification amplifies the effect of contact. Multigroup path analysis revealed that the two stereotype dimensions, warmth and competence, mediate the relationship between interethnic contact and perceived discrimination for Italians, and between organizational identification and perceived conflict for immigrants. Results highlighted an asymmetrical effect of contact on perceived conflict, detrimental and direct for immigrant workers, beneficial and mediate via the outgroup cognitive image for the Italian workers. Findings suggest that a superordinate identity, in terms of organizational identification, may be effective in reducing conflict at the workplace for majority members, whilst a personalized strategy seems to be more suitable for minority members. The theoretical and practical implications of these findings are discussed along with the acculturation perspective.

Migratory flows from the most underprivileged to the wealthiest countries leads to increased diversity in host societies and constitutes a challenge for our new plural societies. Italy, compared to other European countries, is a country of recent immigration but, from 2006 onwards, the annual quota of workers coming from abroad has regularly increased. Factories of North East Italy have recorded a widespread workforce of regular and irregular immigrants at a low qualified professional level. Currently employed in the industry are 18.1% foreign workers and 20.2% Italian workers (Caritas-Migrantes, 2019). This situation has prompted growing interest in finding useful strategies for reducing intergroup conflicts among different ethnic groups.

By examining social psychological theories, such strategies mainly rely on intergroup contact and on the creation of a superordinate common membership. Allport’s (1954) contact hypothesis suggests that, under certain conditions, significant contacts between members of different groups would reduce prejudice towards the outgroup and its behavioral consequences (Hayward, Tropp, Hornsey, & Barlow, 2017; Tropp & Pettigrew, 2005b). On the other hand, the simultaneous salience of multiple dimensions of categorization, and particularly a vertical co-salience of a subordinate and a superordinate group membership (Crisp, Ensari, Hewstone, & Miller, 2002; Dietz, Kleinlogel, & Chui, 2012; Fiol, Pratt, & O'Connor, 2009; Hornsey & Hogg, 2000b), may lead to a restructuring of information processing and ingroup/outgroup evaluations, and play a role in the improvement of intergroup relations.

However, past research has examined the beneficial effect of intergroup contact mainly in majority group members, with a specific focus on the affective dimension of prejudice. Limited research has at the same time considered the effect of intergroup contact in minority and majority group members whilst highlighting cognitive dimensions. To contribute to filling this gap, this study tested whether intergroup contact and organizational identification in a real-world context differentially relates to the perception of interethnic conflict in Italian and non-European Union (N-EU) immigrant blue-collar coworkers. The two ethnic groups, employees of the same factory, are in daily contact and we conjectured that in a workplace context, the cognitive, more than the affective, dimension of prejudice will be the most relevant (Aberson, 2015; Gaunt, 2011). We thus integrated the intergroup contact hypothesis with the stereotype content model (Fiske, Cuddy, Glick, & Xu, 2002), hypothesizing that reciprocal cognitive stereotypes and attitudes operate as potential mediators between intergroup contact, organizational identification and perceived conflict.

## Contact Effects in Minority and Majority Groups

In psychosocial research, decades of studies have suggested that intergroup contact exerts a beneficial effect on intergroup relationships. However, most studies have focused on the majorities or members of the host societies. Less attention has focused on minority status groups, although recent contributions have suggested that in disadvantaged groups the contact effects are weaker (Tropp, 2007; Tropp & Pettigrew, 2005b), nonexistent (Binder et al., 2009) or even detrimental (Hayward et al., 2017; Stephan et al., 2002; Vedder, Wenink, & van Geel, 2017). Ethnic minority group members are generally aware of the negative stereotypes that dominant majority members have of them (Shelton, 2003) and concerned of being discriminated against (Tropp & Pettigrew, 2005b). The expectations of becoming possible targets of prejudice and discrimination may induce negative propensity towards intergroup interactions in minority group members and may reduce the contact effect (Tropp, 2007).

Also when exploring at the same time majority and minority group members (Al Ramiah & Hewstone, 2012; Árnadóttir, Lolliot, Brown, & Hewstone, 2018; Binder et al., 2009; Lutterbach & Beelmann, 2020; Tropp, 2007; Yucel & Psaltis, 2020), research has mainly focused on the mediational role of the affective component of prejudice (Islam & Hewstone, 1993; Stephan & Stephan, 2000; Tropp & Pettigrew, 2005a). In a work context, the cognitive dimensions of prejudice, which fewer empirical studies have examined (Brambilla, Hewstone, & Colucci, 2013; Gaunt, 2011; Kotzur, Schäfer, & Wagner, 2019), are probably more relevant than affective mediators. A great deal of time is spent at the workplace, often engaging with people and establishing instrumental rather than close relationships. These relationships are subject to cogent norms, such as productivity targets, the risk of being made redundant, management demands, competition, cooperation, productivity pressure. Recent demographic factors have, in the meantime, changed the composition of workforce organization, and the study of group-based conflict within ethnically diverse work teams has become an increasingly important focus for contemporary research (Christian, Porter, & Moffitt, 2006; Ensari, 2002; Luijters, van der Zee, & Otten, 2006). Coworkers of different ethnic groups are often in forced daily contact at the workplace, although they can freely choose to engage in other kinds of interpersonal contact. Voluntary or involuntary involvement in contact has also quite a different impact on intergroup relationships (Allport, 1954). Hence, it is relevant to examine also the impact of voluntary or outside-of-work contact, which can take place in cafés, pubs, restaurants, at the gym, etc.

## Organizational Identification

The relationship between an individual and an organization has generally been conceptualized in terms of the individual’s commitment to the organization; however, drawing on the social identity theory (Tajfel & Turner, 1979), a conceptualization in terms of organization identification has also been proposed (Ashforth & Mael, 1989; Hogg & Abrams, 1988). These two conceptualizations are partly overlapping, but organizational identification is more closely linked to individual self-definition (Van Knippenberg & Sleebos, 2006) and therefore may have more relevant consequences on intergroup biases.

In a factory environment, it is particularly evident that group relations take place within the context of a relevant superordinate identity (Hornsey & Hogg, 2000a). Identification with the organization may operate by generating positive views of the outgroup members, underlining their status of coworkers. However, while such a superordinate identity may coincide with subgroup identity for the members of the dominant majority (Kessler & Mummendey, 2001), its excessive underscoring may represent a threat for low-status subgroup members because the superordinate identity is likely to be dominated by the high-status subgroup (Hornsey & Hogg, 2000b). In minorities, protecting their identity distinctiveness may prevail over their need for a superordinate identity as a strategy for identity enhancement (Hornsey & Hogg, 2000a).

Theoretical models on diversity management at work identify as key antecedents of intergroup conflict factors such as social identity and categorization, intergroup contact, realistic group conflict and social dominance orientation, with the mediation of stereotype and prejudice (Dietz et al., 2012). The groups’ different status can lead to stereotype differences connected to a sense of threat/hostility towards the outgroup (Stephan et al., 2002). The perception of unequal distribution of resources, and therefore of perceived discrimination towards the ingroup, may rely on very different aspects and may have different effects in groups with different status, just as their discriminatory behaviors are different (Amiot & Bourhis, 2005). Discrimination refers to an unequal behavior or treatment toward persons or groups due to their affiliation to social groups. However, perceived and experienced discrimination are quite different from the perspective of low-status minority or high-status majority groups (Tajfel & Turner, 1979). For low-status minority members, who feel that they have limited access to the resources society has to offer, the perception of an unfair resource distribution between groups may be particularly salient and therefore may be a relevant factor influencing the perception of conflict, while a wish for an unequal distribution of resources may be particularly relevant for high-status majority members (Amiot & Bourhis, 2005; Esses, Jackson, & Armstrong, 1998) and not a source of conflict. In a work context, a focus on common identity represented by identification with the factory can however reduce the perception of differences in power between groups, and particularly in a disadvantaged group may undermine members’ tendency to make attribution to discrimination (Saguy & Chernyak-Hai, 2012).

## The Stereotype Content Model and Cognitive Mediation

Among the strategies that may be undertaken in an attempt to remove the sources of intergroup conflict, Esses et al. (1998) suggest that one group may try to decrease other groups’ competitiveness. This may take the form of expressing negative evaluations and attributions towards members of other groups, including negative traits. The dimensions on which group members try to differentiate in a positive way may change according to contexts (LeVine & Campbell, 1972) and, in a work setting, judgements of reciprocal competence and warmth may be particularly relevant. For instance, Ensari and Miller (2005) found that both attribution of ability and friendliness to outgroup members are significant mediators of prejudice reductions after an intergroup performance. The model of stereotype content (Fiske et al., 2002) predicts that assessment of other groups’ warmth and competence will emerge from the perception of each group’s level of competition and status, respectively. Specifically, perceived competence is primarily determined by perceived social status and power, whereas perceived warmth is determined by perceived intergroup competition. Thus, people attribute competence to those perceived as high status, and they attribute warmth to those who are not competitive with the ingroup for resources. The first kind of stereotype—competent and cold—proposed by Fiske and colleagues (2002) is defined as envious prejudice, while the second kind—incompetent and warm—is defined as paternalistic prejudice.

Despite the relevance of social structural variables such as competition and status as predictors of warmth and competence outgroup attribution, and ultimately behavioral reactions in intergroup relationships, few studies have linked the intergroup contact hypothesis to the stereotype content models, mainly finding that contact enhances the stereotype content dimension on which a specific outgroup is derogated (Al Ramiah & Hewstone, 2012; Brambilla et al., 2013; Kotzur et al., 2019). According to the logic of the model, we expected that contact affects both warmth and competence perceptions of the two groups involved in our study. However, because of the different status of the two ethnic groups, we expected to find asymmetries in their reciprocal stereotypical content: the Italian workers, the high-status group, would hold a paternalistic stereotype with the attribution of low competence and high warmth to the low-status outgroup, the N-EU immigrants; while the N-EU workers would hold an envious stereotype, with the attribution of high competence but low warmth to the Italian workers (following Fiske et al., 2002).

Based on evidence, we first predicted that both intergroup contact and organizational identifications would increase the knowledge of the outgroup and enhance the perception of competence and warmth of outgroups and improve the attitude towards the outgroup. We further hypothesized that this enhancement would mediate the relationships between contact and organizational identification and perceived discrimination and conflict, albeit differentially in Italian and immigrant worker groups.

## Hypotheses

This study focuses on the perception of interethnic conflict at the workplace both in N-EU immigrant minority and Italian majority members. All participants are blue-collar workers, not often considered in psychosocial studies, despite most ethnic minority members ending up as unskilled factory workers. The amount of voluntary contact, in several situations where the coworkers may meet, and identification with the superordinate identity group, represented here by the firm they all worked for, are expected to be differentially related to perceived conflict in ethnic majority and minority group members, both directly and indirectly, via the cognitive dimension of intergroup relationships (Brambilla et al., 2013; Gaunt, 2011; Kotzur et al., 2019).

In line with the classical psychosocial models of attitudes (Esses, Haddock, & Zanna, 1993), we expected to find a strong relationship between stereotypical content, attitude towards the outgroup, and its behavioral consequences in both ethnic groups, namely perceived discrimination at the workplace, which may in turn increase perceived overt interethnic conflicts at the workplace.

Specifically, our hypotheses are as follows, differentially for the Italian majority and immigrant minority workers:

*H*_1_: As empirical evidence supports that contact has stronger positive consequences on intergroup biases for majority than for minority groups, its effect on perceived conflict was hypothesized to be weaker among the immigrant compared to Italian workers.

*H*_2_: On the basis of previous research regarding the different consequences of superordinate identity on majority and minority groups, Italian workers were expected to have a higher organizational identification, which would be negatively and directly related to perceived discrimination and conflict; whilst immigrant workers were expected to have a lower level of organizational identification, with a weaker and mediated relationship between their organizational identification and perceived discrimination and conflict.

*H*_3_: Following Fiske et al. (2002), considering the different status of the two ethnic groups, we expected to find asymmetries in their reciprocal stereotypical content: the high status group, the Italians, would attribute low competence and high warmth to the low-status immigrant outgroup (paternalistic stereotype), whilst we anticipated that immigrant workers would attribute high competence and low warmth to Italian workers (envious stereotype).

*H*_4_: The stereotype content and the cognitive dimension of the interethnic attitudes drive the indirect effect on the contact/organizational identification and discrimination-conflict relationship.

All hypotheses were tested simultaneously with path analysis models.

## Method

### Participants and Procedure

One hundred and seven blue-collar workers were involved in the study: 67 Italians and 40 N-EU immigrants, all males. The immigrants, working in the same factory and carrying out similar duties, were mostly from Central (17) and North Africa (18), the remainder from Eastern European countries. The age of the respondents varied from 19 to 63 (Age: M = 34.9 years, *SD* = 8.6), with no significant differences between the two groups. On average, the Italians had been working for the firm 3.9 years (*SD* = 0.9) and the N-EU immigrants 3.3 years (*SD* = 0.8; difference not significant at the *t*-test).

Participants were individually approached at the workplace and asked to complete a short questionnaire (approx. 15 minutes), approved by the management. Participants volunteered to complete the survey and signed informed consent.

All factory workers present on the day of the test were contacted and all but a couple of them agreed to participate. The workers did shift work, and 70% of the total 153 workers were on the day shift. Night shifts and day shifts are assigned on a rota system and so the sample was expected to be representative of the whole workforce. Two versions of the questionnaire, for the two groups, were devised in Italian (the immigrants were fluent enough to answer in that language). The measures for both groups were identical except that Italian participants answered questions about immigrant coworkers and vice versa. With adult participants and anonymous questionnaires, ethical approval was not required, in line with national guidelines of the Italian Association of Psychology (AIP).

### Measures

*Outside-of-Work Contact*. To measure the quantity of contact, respondents were asked the following questions adapted from Islam and Hewstone (1993) to the specific context. Five items asked respondents to indicate the frequency with which participants report on their encounters with outgroup members in various non workplace contexts, for example, going to the cinema, or to the pub with Italian/Immigrants coworkers, doing sporting activities together, having dinner, being invited to others’ homes (response options were *never, sometimes, often*). These five items were added up (α = 0.76).

*Organizational Identification*. We adapted two items: “I feel I am part of the factory,” and “I don’t feel particularly respected by the organization” (reversed) (from 1 = *not at all* to 5 = *very much*) from the Organizational Identification Questionnaire (Downs, 1994) Responses were averaged (*r* = .74).

*Stereotypical Content*. Following Fiske et al. (2002), a selection of five traits regarding *competence* (able, intelligent, reliable, responsible, independent; IT α = 0.81; IMM α = 0.80) and *warmth* (friendly, generous, pleasant, modest, patient; IT α = 0.87; IMM α = 0.78) were considered. Participants were asked to rate on 5-point scales the extent to which they personally associated the traits to outgroup coworkers (from 1 = *not at all associated* to 5 = *very much associated*). Two indices were computed from the average.

*Attitude towards the outgroup* was measured on a 10-point feeling thermometer (where 1 = *completely against* and 10 = *completely in favor of Italian/Immigrant coworkers*).

*Perception of discrimination at the workplace* was measured with a single item: “the Italian workers/the N-EU immigrant workers get more than they deserve” (on 5-point Likert scales from 1 = *do not agree at all* to 5 = *strongly;* following Tropp & Pettigrew, 2005a).

*Perception of interethnic conflict* at the workplace was measured with three items: “Is there conflict between Italian and N-EU immigrant workers in the factory?” “On the whole, relationships between Italian and immigrant workers are good” (reversed) (from 1 = *totally false* to 5 = *totally true*); “How often does it occur?” (from 1 = *never* to 5 = *very often*). Responses were averaged to obtain a global index (IT α = 0.75; IMM α = 0.71).

### Data Analysis

Firstly, a MANOVA with Ethnicity as the between factor was conducted on all study variables. Secondly, we explored the antecedent of the perceived conflict in Italians and immigrants with multigroup path analyses (Lisrel 10.2). Typically, multiple items or measures are used to assess latent variables (i.e., measurement model). In the present study such an approach would have produced an unacceptably high ratio of estimated parameters compared to sample size; therefore, the tested models used composite variables of the constructs as observed variables. Hypothesized predictors were: organizational identification and interethnic contact as exogenous variables; reciprocal stereotypical content, attitude towards the outgroup, and perceived discrimination as mediator variables; finally, perception of conflict as outcome variable.

## Results

### Preliminary Analyses

The mean, standard deviations, and correlations for Italian and N-EU immigrant workers are presented in Table 1. The MANOVA on the study variables revealed a main effect of Ethnicity, λ = 0.55, *F* (7, 99) = 11.9, *p* < .001, η_p_^2^ = 0.46. Significant multivariate effects were followed up using appropriate univariate tests (*t*-test). The immigrant workers attributed significantly more competence to the Italian coworkers than their Italian counterparts, *t* (105) = 5.9, *p* < .001, while no differences emerged for warmth that the two groups attributed to each other. However, a pairwise comparison of intragroup attribution showed that the immigrant workers judged Italian coworkers more competent than warm, *t* (39) = 5.44, *p* < .001, while the difference of the warmth and competence attributed to the immigrants group by their Italian coworkers, albeit congruent with expectations, was not significant, *t* (66) = 2.03, ns. As predicted, Italian workers identified significantly more with the firm they work for, *t* (105) = 2.73, *p* = .007, whereas immigrant co-workers reported significantly more frequent contacts with outgroup members, *t* (105) = 3.8, *p* < .05. No significant differences emerged regarding attitude towards the outgroup, perceived discrimination and interethnic conflict at the workplace.

**Table 1:** Means, Standard Deviations, and Intercorrelation for Italian and N-EU Immigrant Workers

Measure	1	2	3	4	5	6	7
1. Organizational identification		.02	.47**	.42**	.50***	−.34*	−.34**
2. Interethnic contact	.10		.10	.09	.09	.09	.20
3. Competence of the outgroup	.29**	.31*		.63***	.43**	−.37*	−.39**
4. Warmth of the outgroup	.26*	.24*	.77***		.54***	−.40**	−.10
5. Attitude towards the outgroup	.22	.14	.31*	.38**		−.48**	−.37**
6. Discrimination at the workplace	−.12	−.05	−.46***	−.45***	−.49***		.51***
7. Conflict at the workplace	−.27*	−.03	−.09	−.17	−.07	.20	
Italian workers Mean (*SD*)	4.03 (1.2)***	6.37 (1.9)*	2.84 (0.6)***	3.07 (0.7)	6.06 (2.4)	2.97 (2.4)	2.82 (1.1)
Immigrant worker Mean (*SD*)	3.38 (1.4)	7.33 (1.8)	3.53 (0.6)	3.01 (1.3)	6.31 (2.9)	2.98 (0.9)	3.10 (1.0)

*Note*. Correlations above the diagonal are for the NE-Immigrants; correlations below the diagonal are for the Italians.

*p < .05. **p < .01. ***p < .001. The asterisks for the means refer to the results of the *t*-test, comparing Italian workers with Immigrant workers.

### Antecedents of Interethnic Conflict

We analyzed the input data in two steps. First, we tested the hypothesized mediation separately in the two groups because antecedents were expected to differentially influence perceived conflict in the Italian and N-EU immigrant workers. Second, multigroup path analyses were employed to examine equality/differences of the paths in the two ethnic groups; details are presented in a supplementary file.

We first tested a model where organizational identification and contact were allowed to predict the component of the outgroup cognitive image (warmth, competence, attitude towards the outgroup), each image variable was allowed to predict discrimination and conflict, and discrimination was allowed to predict conflict. Subsequently, we compared the described *full mediated model*, which included only indirect paths from antecedents to perceived conflict through outgroup image, with a *partial mediated model,* which also included the direct paths connecting organizational identification and interethnic contact with perceived conflict, each added separately.

The models were refined by removing non-significant paths (conventionally *t* < 1.96), as suggested by the Wald test. On the basis of the modification indices, the errors of competence and warmth were allowed to correlate (the correlated measurement errors were assumed to be due to the shared method variance as these constructs were measured on the same scale).

The *partial mediated model,* which adds one direct significant path in each sample, running respectively from organizational identification to conflict for the Italians and from contact to conflict for the Immigrants, fitted the data better (Immigrants: χ*^2^* = 10.74, *df* = 12, *p* = ns, CFI = 1, RMSEA = 0, AIC = 56.7, CAIC = 118.5; Italians: χ*^2^* = 7.37, *df* = 10, *p* = ns, CFI = 1, RMSEA = 0, AIC = 59.2, CAIC = 142.5) than the *fully mediated model* (Immigrants: χ*^2^* = 14.96, *df* = 11, *p* = ns, CFI = 0.92, RMSEA = 0.09, AIC = 61.2, CAIC = 120.4; Italians: χ*^2^* = 10.65, *df* = 11, *p* = ns, CFI = 0.90, RMSEA = 0.03, AIC = 60.7, CAIC = 150.2). The *partial mediation models* had the lowest χ*^2^*/*df* ratio (0.89 and 0.73 respectively vs. 1.36 and 0.97), as well as the lowest RMSEA, AIC and CAIC. We also ran a Chi-square difference test, frequently used to test differences between nested models, that is, two identical models one of which could be obtained simply by fixing/eliminating parameters in the other model. If the χ^2^ diff-value is significant, the “larger” model with more freely estimated parameters fits the data better than the “smaller” model in which the parameters are fixed. Results confirmed that the partial mediated models, with additional direct paths between the predictors and the dependent variables, fit the data better of the full mediated model (Immigrants: Chi-square difference 4.22(1), *p* < .05; Italians: Chi-square difference 3.28(1), *p* = .07).

These partial mediated models were used as a starting point for multigroup path analyses. The final best fitting model emerged by imposing cross-group equality constraints on four parameters in which the partial models of the two ethnic groups have significant similar path (four paths: from organizational identification to warmth and to competence; from warmth to attitude towards the outgroup; from attitude to discrimination), while the other path coefficients were allowed to vary freely across the two samples (χ*^2^* = 19.63, *df* = 27, *p* = ns, CFI = 1, RMSEA = 0, AIC = 107.63, CAIC = 269.24).

**Figure 1:**
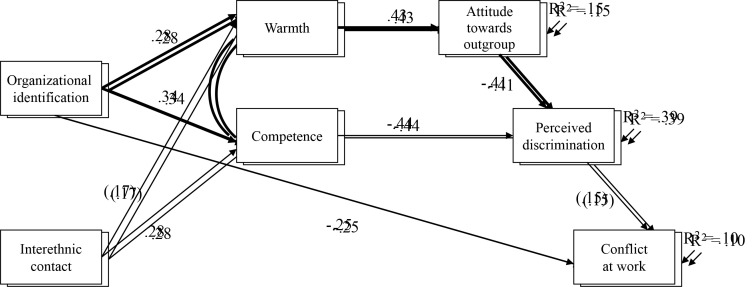
ITALIANS: Multigroup Partially Mediated Path Model *Note*. With the two predictors, indirect effect and perceived conflict, as outcome variable. Only significant paths are reported (*T* > 1.96, *p* < .05; parenthesized values marginally significant). Coefficients are standardized betas. Curved lines indicate error covariances. Paths constrained to be equal in the two samples are shown as thicker lines.

**Figure 2:**
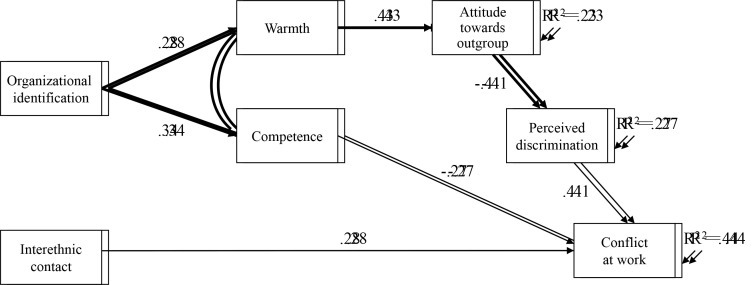
Non-EU IMMIGRANTS: Multigroup Partially Mediated Path Model

The estimated path coefficients are presented graphically in Figure 1 for Italian workers and in Figure 2 for immigrant workers. The four causal paths equivalent across both samples (represented by the thicker lines) were significant and as expected: organizational identification showed a positive relationship with a better stereotypical content in terms of warmth and competence attributed to outgroup members; the attribution of warmth had a positive direct association with a favorable attitude towards the outgroup, which in turn was directly significantly and negatively related to discrimination at the workplace, in line with the classical psychosocial model of attitude.

However, some notable differences emerged between the two samples. In the Italian group, contact was related to an improved outgroup image, particularly in terms of the competence attributed to the ethnic coworkers. In turn, competence had a negative direct association with perception of discrimination at the workplace. Discrimination was positively related to perceived conflict for both samples, albeit somewhat weaker for the Italians, where it did not reach significance. The only significant direct association with perceived conflict is identification with the superordinate ingroup. The amount of variance explained (squared multiple correlation *R*^2^) was 15% for ethnic attitude, 39% for perceived discrimination, 10% for perceived conflict.

The model for the N-EU immigrants no longer showed any direct association between organizational identification and perceived conflict. Moreover, the attribution of competence to the Italian coworkers had a direct negative association with perceived conflict, and the relation between discrimination and perceived conflict was significant. Therefore, in the case of immigrant workers, the image of the outgroup coworkers significantly mediated the relationship between organizational identification and perception of discrimination and conflict at the workplace. Interethnic contact is directly and negatively associated with perceived conflict. The amount of variance explained was 23% for ethnic attitude, 27% for perceived discrimination, and 44% for perceived conflict.

## Discussion

The study focused on perceived interethnic conflict at the workplace in Italian and N-EU immigrant blue-collar coworkers. Findings support our general hypothesis that interethnic contact and organizational identification differentially influence perceived interethnic conflict in the two ethnic groups with the mediation of the cognitive dimension of interethnic relations.

Supporting and extending prior research on its lesser impact among disadvantaged groups (Hayward et al., 2017; Stephan et al., 2002; Tropp, 2007; Tropp & Pettigrew, 2005b; Vedder et al., 2017), among the immigrant workers the effect of interethnic contact was not only weaker, as expected (*H*_1_), but rather detrimental as it is directly and positively associated to the perception of conflict. On the contrary, among Italians, it has a beneficial albeit indirect effect, improving the outgroup’s stereotypical image. A possible explanation may be that negative interethnic contact is more prominent in the minority than in the majority workers (Barlow et al., 2012; Vedder et al., 2017). Negative contact has been found to be directly related to negative attitude (Pettigrew & Tropp, 2006; Tropp & Pettigrew, 2005b), and positive-negative asymmetry was observed (Barlow et al., 2012), with negative interactions which may be more influential than positive ones on intergroup biases, thus neutralizing the effects of many positive contacts (Árnadóttir et al., 2018; Hayward et al., 2017; Meleady & Forder, 2019; Stephan et al., 2002; Vedder et al., 2017). Also, Aberson (2015) found that negative contact more strongly predicted cognitive dimension of prejudice, probably increasing the salience of group membership, while positive contact predicts affective dimensions but does not consistently relate to the cognitive dimension (Paolini, Harwood, & Rubin, 2010). In a work context, interactions may often be negative (Ensari & Miller, 2006) because of competition within the setting, pressure to produce, supervisors’ controls, work stress in general. Additionally, when competition over resources arises, proximity and contact may increase rather than decrease intergroup hostility (LeVine & Campbell, 1972).

Our expectations that among the Italian workers, identification with the superordinate ingroup is higher and directly and negatively associated with the perception of interethnic conflict (*H*_2_) were also confirmed. Asymmetries in the stereotype content only partially confirm our hypothesis (*H*_3_): the low-status N-EU immigrant workers recognize greater competence to the high-status Italian workers, although the latter do not attribute higher warmth to the low-status immigrant workers. Therefore, the N-EU Immigrant workers hold an envious prejudice but the Italian workers do not adhere totally to a paternalistic prejudice (Fiske et al., 2002).

On the whole, the classical relationships between stereotype, attitude, and its behavioral consequences, that is discrimination, have been confirmed in both groups. Specifically, the attribution of greater warmth is positively related to the attitude towards the outgroup colleagues and indirectly to perceived discrimination; the attribution of higher competence to the outgroup may reduce perceived discrimination in the Italian workers and perceived conflict in the Immigrant workers.

The two dimensions of the stereotype, warmth and competence, mediate the relationship between interethnic contact and perceived discrimination for the Italians, and between organizational identification and perceived conflict for the immigrants (confirming *H*_4_). Cognitive prejudice measures, like those used in the current study, reflect participants’ beliefs about the outgroup and may exert a relevant role in specific circumstances and specific behavioral consequences such as perceived discrimination and conflict at the workplace, due to the establishment of instrumental more than close relationships.

No significant difference in the amount of perceived discrimination and conflict in the two groups emerged, in line with past evidence supporting the absence of systematic relationships between group status and intergroup biases in real-world groups (Mullen, Brown, & Smith., 1992). An important condition in intergroup relationships is whether social groups find themselves (or believe they find themselves) in competition because of scarce resources. According to the realistic conflict theory, it is the perception of competition and threat, more than actual competition over resources, that leads to prejudice and intergroup conflict (Esses et al., 1998; Stephan & Stephan, 2000). The perception of unequal distribution of resources, and therefore of perceived discrimination towards the ingroup, may rely on very different aspects in groups with different social status (Amiot & Bourhis, 2005; Esses et al., 1998). Our findings support this view: when the Italian workers recognize greater competence in their immigrant colleagues, there is less discrimination, but this does not reduce perceived conflict; in the N-EU immigrant workers, recognizing competence of their Italian colleagues directly decreases perceived conflict. Perceiving substantial discrimination contributes to the prediction of interethnic conflict in minority group members, while such perceptions are almost unrelated among majority members; similarly, perceived discrimination has been found to moderate the relationship between interethnic contact and improved intergroup closeness particularly for members of disadvantaged groups (Tropp, 2007).

In both ethnic groups, organizational identification may reduce conflict, directly for the Italian workers and with the mediation of outgroup stereotypical image for the N-EU immigrant workers. A crucial variable for increasing intergroup harmony is therefore the extent to which specific context facilitates the development of a superordinate identification. Emphasis on a single, inclusive group may alter the conceptual representation of group members, and employees might focus on their shared organizational identities (Dietz, et al., 2012). But, as Hornsey and Hogg (2000a) remark, minority-group individuals in a majority-dominated organization may feel their identity threatened in a context which stresses exclusively organizational identification, provoking behavior aimed at protecting subgroup social identity. This defensive reaction, besides having other effects on ingroup biases and conflict, may reduce superordinate group identification in low-status group members (Dovidio, Gaertner, & Validzic, 1998). Indeed, our immigrant workers reported lower levels of organizational identification. As underlined in literature, increasing the salience of a pre-existing superordinate identity should be achieved in a context where group members retain a distinct identity. The “simultaneous categorization” (Hornsey & Hogg, 2000b) approach can yield more positive effects on interethnic conflict reductions than the complete abandonment of subgroup identities.

Approaches within the acculturation framework propose similar explanations of bias reduction (Berry, 1997). Our findings suggest that the Italian workers perceived less conflict to the extent that they were identified with the organization, whereas indicators of cultural respect and acceptance (e.g., attitude, perceived discrimination, contact) predicted perceived conflict for the immigrants. This may reflect assimilationist expectations in the majority group but integration/multiculturalist expectations in the minority group. In the immigrant worker group, contact may encourage expectations of integration and acceptance that do not materialize as quality of interactions both in and out of the work setting. Generally, ethnic minorities favor integration over assimilation, whilst the dominant majority tends to favor assimilation (Verkuyten, 2005). Research has consistently shown that ethnic minorities respond most positively to the integration strategy (preserving original cultures and habits while acquiring some characteristics of the host culture, in contrast to assimilation), and that this strategy predicts greater psychological and sociocultural adaptation and overall well-being of minority-group members (Berry, 1997; Berry, Phinney, Sam, & Vedder, 2006; LaFromboise, Coleman, & Gerton, 1993). Luijters and colleagues (2006) tested preferred acculturation strategy at the workplace and concluded that dual identity is the most positively evaluated strategy among ethnic minority workers. Within the framework of the MIRIPS (Mutual Intercultural Relations in Plural Societies), an Italian study (Inguglia et al., 2020) compared immigrant and Italian adolescents and showed that in the non-dominant group, contact with Italian peers was associated with integration while, in line with our results, perceived discrimination with separation and lower psychological well-being.

However, as Berry (1997) affirms, multiculturalism is a strategy that can also yield stress when the majority group is not open and inclusive. Lack of fit between majority and minority acculturation expectations generally leads to worsening intergroup relations (Zagefka & Brown, 2002). If this is true and hardly modifiable in a wider social context, in a more limited and easily controlled working set a feasible objective could be that of making the acculturation orientation of workers of diverse ethnic groups coincide within a framework of integration support on the part of management.

The study has limitations. Firstly, it relies on a limited sample size, mainly due to the circumscribed population of the specific real-world context involved. Secondly, in a correlational study it is impossible to determine the direction of causality. In Stephan and Stephan’s (2000) integrated threat model, perceived conflict is conceptualized as an antecedent of ethnic attitude, while here it is argued to be a consequence of it, and our findings support this view, especially as far as the minority group is concerned. The most accurate description may not be a linear process, but rather a circular trend in which some conditions produce conflicts, thus affecting attitude. Finally, we did not measure the positive or negative quality of contact, which may be usefully addressed in future studies, both for involuntary contact at work and voluntary outside-of-work contact.

Despite these limitations, our conclusions are based on a field study with real-group interactions, with the concurrent use of coworkers of both majority and minority groups. Reducing interethnic conflict at the workplace is an extremely relevant task for the management (Ensari, 2002), and our findings show that the factors underlying perception of conflict in minority and majority workers are different and call for different strategies.

Stressing identification with the factory they all work for may be effective for majority members, given that they are not likely to feel their subgroup identity threatened as are members of the majority group (Kessler & Mummendey, 2001), whereas it may be less effective for minority members (Hornesy & Hogg, 2000a). It would probably be better to start with personalization (Ensari, 2002): management should create more occasions for initiating and developing personalized contact, trying to improve both the amount and quality of interethnic interactions in and out of the factory. The aim is acting on minority-group workers’ perception of being discriminated, strongly associated with perceived conflict, by reinforcing the salience of common goals, recognizing reciprocal competence, fostering cooperation between heterogeneous workgroups (Chrobot-Mason & Ruderman, 2004). This bottom-up process, where the information encoded about the outgroup member during social interaction is dominated not by the relevant social category, but by the unique qualities of that individual (Ensari, Christian, Kuriyama, & Miller, 2012; Ensari & Miller, 2006), can make cognitive representations of the outgroup more complex. Our findings highlight that for ethnic minority workers the beneficial effect of the organizational identification is mediated by an improved image of the Italian co-workers. A gradual combination of decategorization/personalization strategies and recategorization may therefore be beneficial for minority members (Ensari & Miller, 2006), additionally so if this is matched with a reduction of potential negative consequences of contact at intergroup level. Finally, keeping distinct group identities and contemporarily reinforcing the salience of common membership, are psychosocial models proposed for prejudice reduction compatible with multiculturalism. Intergroup research and acculturation perspectives may be usefully combined in order to better comprehend conflict reduction in work contexts.

In conclusion, as observed by Shelton (2000), research should try to understand how ethnic minorities’ attitudes and behaviors affect quality of interactions with the majority group. Our findings emphasize that what is known about majority groups is in no way automatically applicable to a minority group, and the importance of comparing minority and majority group members in real world situations.

## Supplementary Materials

For this article the following Supplementary Materials are available via PsychArchives (for access see Index of Supplementary Materials below):

- Appendix: Antecedents of interethnic conflict – Alternative path models.

MonaciM. G.
 (2022). Supplementary materials to "Interethnic workplace conflict: Reciprocal perception of italian and immigrant blue-collar coworkers"
[Appendix]. PsychOpen. 10.23668/psycharchives.6697
PMC963254936348694

## Data Availability

The raw data will be made available by the authors, without undue reservation, to any qualified researcher upon request.

## Supplementary Materials

####index will be inserted#####
